# Gender Differences in Outpatient Pediatric Drug Utilization: A Cohort Study From Southern Italy

**DOI:** 10.3389/fphar.2019.00011

**Published:** 2019-02-05

**Authors:** Carmen Ferrajolo, Janet Sultana, Valentina Ientile, Cristina Scavone, Giulia Scondotto, Michele Tari, Gianluca Trifirò, Francesco Rossi, Annalisa Capuano

**Affiliations:** ^1^Clinical Pharmacology Unit, Department of Experimental Medicine, University of Campania “Vanvitelli”, Naples, Italy; ^2^Center of Pharmacovigilance and Pharmacoepidemiology, Campania Region, Naples, Italy; ^3^Department of Biomedical and Dental Sciences and Morphofunctional Imaging, University of Messina, Messina, Italy; ^4^Clinical Pharmacology Unit, Azienda Ospedaliera Universitaria Policlinico “G. Martino”, Messina, Italy; ^5^Local Health Unit of Caserta, Caserta, Italy

**Keywords:** gender, drug utilization, pediatric, outpatient, antibiotics, respiratory tract drugs, systemic corticosteroids

## Abstract

**Objective:** The aim of this retrospective population-based cohort study is to in-depth investigate gender-specific drug utilization pattern in pediatric outpatient population.

**Methods:** By using a large administrative database of the Local Health Unit of Caserta (Southern Italy), a pediatric cohort from the birth to 18 years was observed over 6 years (from 1st January 2010 to 31st December 2015). Yearly prevalence of drug use per 100 inhabitants as well as the median number of prescriptions was stratifying by gender. Prevalence of acute and recurrent use of the most frequently used active substances was calculated for the year 2015.

**Results:** A decreasing trend in prevalence of drug use (−3.2%, with a reduction of median number of drugs dispensed) was observed in children for both sexes, from 2010 to 2015. In 2015, the drug classes most commonly used among children of any age were modestly but consistently prescribed more to males than to females: systemic anti-infective drugs (*M* = 43.5%; *F* = 42.3%), respiratory tract drugs (*M* = 29.0%; *F* = 26.1%), and hormones (*M* = 13.1%; *F* = 11.3%). Irrespective of gender, beclomethasone was the most utilized active substance in the first 2 years of life, while thereafter amoxicillin/clavulanate in combination.

**Conclusions:** In a large population of pediatric outpatients no major difference was seen between genders, although commonly used drug classes; in particular, antibiotics, respiratory tract drugs and Hormones with corticosteroids for systemic use prescribed modestly but consistently to larger extent in males than females.

## Introduction

The effectiveness and safety of medicines in children are largely deduced from clinical trials for adults. However, such extrapolation is limited by the nature of pharmacokinetic and pharmacodynamic parameters in children, which are very different compared to adults (Van Den Anker and Allegaert, 2012). Moreover, children cannot be considered a single homogeneous group, as there are significant variations between neonates, infants, children, and adolescents in terms of pharmacokinetics and pharmacodynamics (Van Den Anker and Allegaert, 2012). As a result, off-label or otherwise unlicensed use of drugs in children is common, leading to an increased risk of potentially serious adverse reactions (Schirm et al., 2003; Clavenna and Bonati, 2009a). This highlights the importance of investigating drug use in pediatric patients. Several initiatives were implemented at the international and national level to address medicine-related issues among children, such as the creation of the Pediatric Committee within the European Medicines Agency (EMA), the implementation of Pediatric Investigation Plans, research plans required by EMA aimed at ensuring that clinically important medicine-related pediatric data is available, and the Pediatric-Use Marketing Authorization, which is a pediatric-specific marketing authorization, also required by (European Committee, 2006). In addition to these initiatives promoting an increase of clinical trials in children, the growing amount of real-word pediatric data is a valuable tool to quickly provide evidence on the use and safety of medicines among children. Indeed, increasing knowledge on pediatric drug use is a potentially important first step to optimize drug utilization in children. A review of 11 observational studies evaluating drug prescribing in pediatric populations in developed countries suggests that children are widely exposed to drugs, with an estimated prevalence of 60% worldwide (Clavenna and Bonati, 2009b), although more recent data from US indicates a significant decreasing trend in drug prescription in this population (Chai et al., 2012). Interestingly, among the youngest age groups, medicines, particularly anti-infective and respiratory tract agents, were more commonly prescribed to boys than girls. This pattern was reversed in adolescence, when the prevalence of almost all drug classes (except non-sex hormones) was higher among girls than boys (Clavenna and Bonati, 2009b). Moreover, similar results were provided by Clavenna et al. when the use of medicines was explored in Italian children in outpatient setting (Clavenna et al., 2009b). The overall trend of higher prevalence medicine use among boys than girls, except for population aged 14–17, was confirmed in the National Report on Medicines use in Italy (OsMed, 2015).

In addition, it has been suggested that higher drug exposures among males than females may explain higher reporting rates of adverse drug reactions (ADRs) among them (Hohmann, 1989; Zopf et al., 2009). In fact, recent observational studies of national and international spontaneous reporting systems of ADRs in children consistently showed a greater prevalence of ADR reports among pre-adolescent boys than girls, while this trend reversed during adolescence, with a higher occurrence of ADRs among girls aged 14–17 than boys (Star et al., 2011; Ferrajolo et al., 2014).

Evidence from scientific literature suggests that children in Italy are exposed to more drugs on average, as compared to other populations, thus calling for more careful post-marketing assessment of medicine use. In particular, the frequency of exposure to antibiotic drugs among Italian children has been estimated 4-fold higher than among English children and 3-fold higher than among Dutch children (Sturkenboom et al., 2008).

Despite the need to shed light on this matter, there are no recent studies evaluating gender-based differences drug utilization in detail in a pediatric setting in large populations, including Italy. The aim of this retrospective population-based, drug utilization study was to investigate gender-specific drug utilization patterns in a pediatric population of large geographic area from Southern Italy.

## Materials and Methods

### Data Source

This retrospective cohort study was conducted using record linkage of claims databases from the Local Health Unit (LHU) of Caserta from Campania region (Southern Italy). The source population consists of 1,118,355 residents living in catchment area of Caserta. Claims databases contain anonymized data of patient demographics, drug dispensing (hereafter referred to as prescriptions), hospital discharge diagnoses, and mortality and disease registries. Dispensing data include information on active ingredient, classified using the Anatomical Therapeutic Chemical (ATC) classification system, the quantity of packages dispensed and the dispensing date. Dispensing information concerns drugs which are reimbursed by the Italian National Health System (NHS), prescribed mainly by the family pediatrician (FP)/general practitioner (GP) or specialists working in the public or private sector. In Italy the NHS provides every child up to the age of 14 with medical care by a FP, while from 14 years of age onward, the child's family can choose a GP for routine care. The dispensing database also captures information on drugs dispensed in other ways, for example, drugs directly supplied to patients by the LHU (known as Direct Distribution), or by hospitals to outpatients through local pharmacies.

This study was part of the “*Medicine Use and Safety in Children*—MUSiC” project, funded by the Italian Ministry of Education, University and Research, under Scientific Independence of young Researchers (SIR) program (No. RBSI14X2S5). No ethics committee approval is required to use anonymous patient data which is analyzed retrospectively.

### Study Population

All inhabitants registered in the Caserta LHU databases who were under 18 years during the period from 1st January 2010 to 31st December 2015 were considered eligible for inclusion in this study, as done in previous studies (Clavenna et al., 2009b; Piovani et al., 2013). The pediatric cohort included male (M) and female (F) neonates (age < 28 days), infants (age 28 days to 2 years), children (age 2 to 11 years) and adolescents (age 12 to 17 years) who had a database history of at least 6 months or who were born during the study period. Each child started contributing to the study from the start of study period (1st January 2010) or the date of registration in the database or date of birth, if occurring during the study period, till the end of study period (31st December, 2015) or date of transferring out or the 18th birthday or death, whichever came first.

### Data Analysis

Drug utilization in the pediatric outpatients was initially described in terms of prevalence of use and number of prescriptions without specifying drug class over time of study period. The yearly prevalence of use per 100 inhabitants (with 95% confidence interval, CI) was calculated from 2010 to 2015 by dividing the number of children from Caserta catchment area with at least one prescription by the total number of children resident in the same area during the study period, stratifying by gender. The difference in prevalence per 100 children was estimated using the chi-squared test for trend. The number of prescriptions was reported as medians (along with inter-quartile range).

Further analyses were conducted only in 2015 as this is the most updated clinical data available. The prevalence of use of the most frequently prescribed medicines in children on 2015 was estimated per 100 inhabitants, stratifying by gender and age group, at different ATC level: therapeutic drug classes (1st level), therapeutic subgroups (2nd level), and active substances (5th level). Then, as an indicator of recurrent/chronic use of an individual compound, the proportion of children with at least 3 prescriptions in 1 year of observation (i.e., 2015) of that specific compound has been stratified by age-category and gender. Moreover, looking at the mostly used drug classes, the recurrent use was estimated also within antibiotics (2nd level ATC J01), corticosteroids for systemic use (H02) and, separately, for β_2_-agonist agents (4th level ATC: R03AC), inhaled corticosteroids (R03BA), and their combinations (R03Ak or R03AL). In addition, gender difference was evaluated even for children concerning polytherapy, i.e., treated concomitantly with at least four active substance (5th level of ATC). The overlapping dates of four or more prescriptions have been defined as concomitant use of medicines.

As secondary analysis, we calculated defined daily dose (DDD) per 1,000 persons per day to evaluate the gender-distribution of the overall consumption of medicines used in children with the final goal to compare our results with data coming from National Report on Medicines use in Italy (OsMed, 2015). The distribution of DDD/1,000 persons per day was stratified by males and females.

All data management and statistical analyses were conducted using SAS version 9.4. *P*-values below 0.05 were used to denote statistical significance.

## Results

Using the source population of 1,118,355 residents, a cohort of 283,878 patients (25.4%) younger than 18 years was identified between 2010 and 2015.

As shown in Table 1, the yearly prevalence of drug use per 100 children was slightly but significantly decreasing during the study period, varying from 56.7% (CI 95%: 56.5–56.9) in 2010 to 53.5% (53.3–53.8) in 2015 (*p* < 0.001). Consistently, the median number of prescriptions per child showed a slight decrease, from 3 (interquartile range: 1–6) to 2 (interquartile range: 1–4) during the observation years. Specifically, we observed relevant change in the number of antibiotic prescriptions, reduced from 48.9% (49.1–48.7) in 2010 to 42.9% (43.1–42.7) in 2015 (data not shown). Stratified analysis by gender showed an overall prevalence of drug use modestly and constantly higher in males than females.

**Table 1:** Characteristics of the study population and prevalence of drug use per 100 inhabitants during 6 years of observation period.

**Study year**	**2010**	**2011**	**2012**	**2013**	**2014**	**2015**	***p*-value**
**Total**	202,556	202,132	199,752	196,070	192,212	188,197	–
No. of treated	114,847	113,402	108,245	109,043	106,253	100,730	< 0.001
Prevalence of drug use/100 children (95% CI)	56.7 (56.5–56.9)	56.1 (55.9–56.3)	54.2 (54.0–54.4)	55.6 (55.4–55.8)	55.3 (55.1–55.5)	53.5 (53.3–53.8)	–
No. of prescriptions	507,268	474,838	441,661	458,507	448,785	372,922	< 0.001
Median number of prescriptions per child (IQR)	3 (1–6)	3 (1–5)	3 (1–5)	3 (1–5)	3 (1–5)	2 (1–4)	–
**Female (% of total)**	98,474 (48.6)	98,266 (48.6)	97,110 (48.6)	95,383 (48.7)	93,430 (48.6)	91,526 (48.6)	–
No. of treated	55,071	54,356	51,714	52,183	50,835	48,217	< 0.001
Prevalence of drug use/100 children (95% CI)	55.9 (55.9–56.2)	55.3 (55.0–55.6)	53.3 (52.9–53.6)	54.7 (54.4–55.0)	54.4 (54.1–54.7)	52.7 (52.4–53.0)	–
N. of prescriptions	232,917	219,061	202,202	209,749	205,000	171,109	< 0.001
Median number of prescriptions per child (IQR)	3 (1–5)	3 (1–5)	2 (1–5)	3 (1–5)	3 (1–5)	2 (1–4)	–
**Male (% of total)**	104,082 (51.4)	103,866 (51.4)	102,642 (51.4)	100,687 (51.3)	98,782 (51.4)	96,671 (51.4)	–
No. of treated	59,776	59,046	56,531	56,860	55,418	52,513	< 0.001
Prevalence of drug use/100 children (95% CI)	57.4 (57.1–57.7)	56.9 (56.6–57.2)	55.1 (54.8–55.4)	56.5 (56.2–56.8)	56.1 (55.8–56.4)	54.3 (54.0–54.6)	–
No. of prescriptions	274,351	255,777	239,459	248,758	243,785	201,813	< 0.001
Median number of prescriptions per child (IQR)	3 (1–6)	3 (1–5)	3 (1–5)	3 (1–5)	3 (1–5)	2 (1–5)	–

*IQR, interquartile range; CI, confidence interval*.

**Figure 1:**
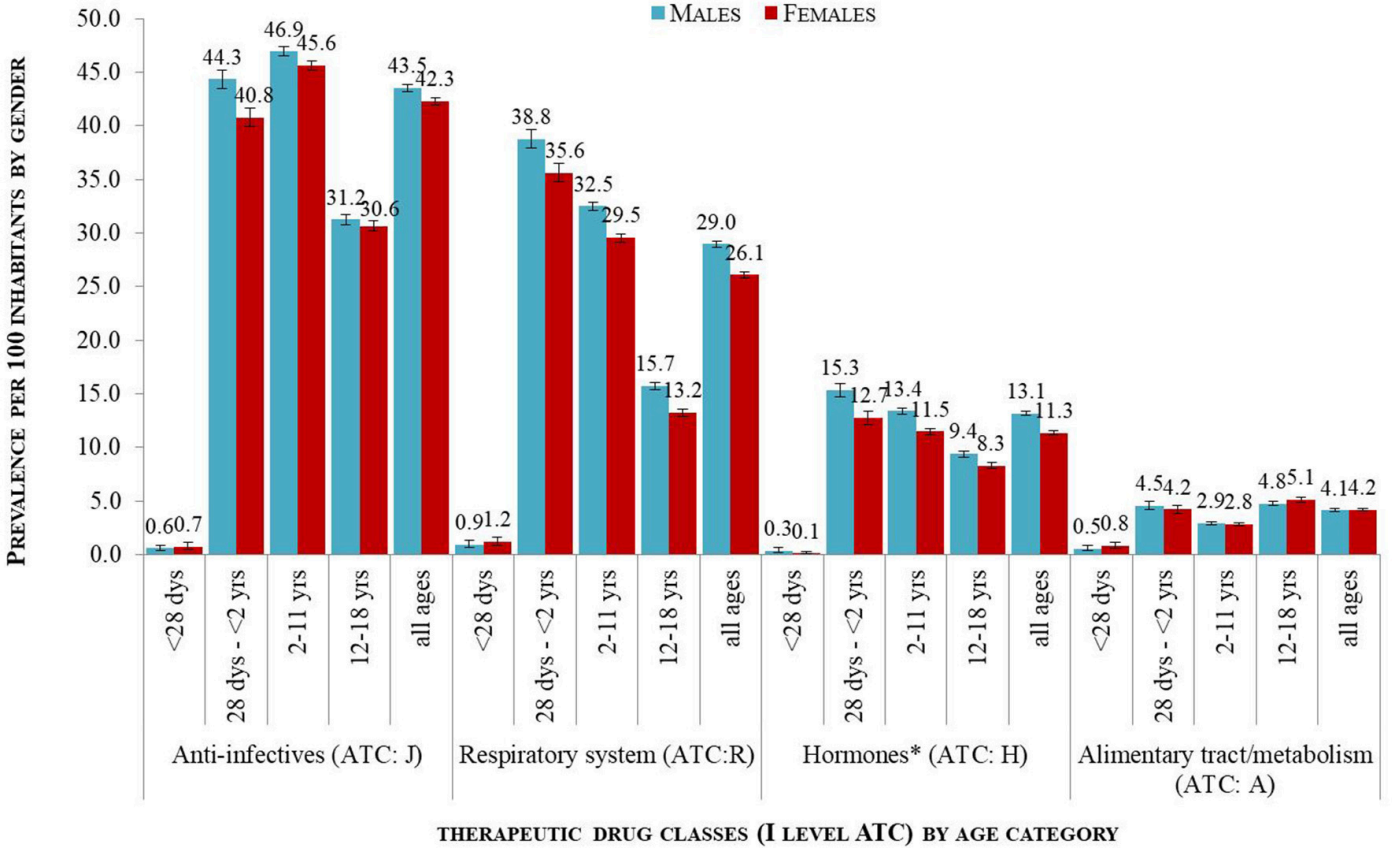
Prevalence per 100 children distributed by age groups and gender by 1st level of ATC for the most commonly used drug classes in 2015. ^*^Included systemic hormonal preparations and excluded sex hormones and insulins.

Focusing on pediatric prescriptions from 2015 the analysis confirmed the main results. Looking at the most commonly used drug classes (1st level ATC) among the total pediatric population (from birth to 18 years), the prevalence was almost always slightly but statistically significantly higher for males than females. As shown in Figure 1, small gender-difference was observed for systemic anti-infective drugs (1st level ATC J^*^: M = 43.5%; F = 42.3%), respiratory tract drugs (R^*^: M = 29.0%; F = 26.1%), hormones (H^*^: M = 13.1%; F = 11.3%), while not for drugs relating to the alimentary tract/metabolism (A^*^: 4% of males and females). Stratifying by age-category, the prevalence still remained slightly higher in males; of note, the prevalence of use of anti-infective agents, respiratory tract drugs and hormones a significantly decreased among adolescents (12 years or older) likewise for both sexes. Specifically, among systemic anti-infective drugs, the most striking gender differences were seen for infants (from 28 days to 2 years) (M = 44.3%; F = 40.8%). For respiratory tract drugs on the other hand, more modest gender differences were observed in all the age groups investigated, that is, among children aged 28 days to 2 years (M = 38.8%; F = 35.6%), 2 to 11 years (M = 32.5%; F = 29.5%) and 12 to 18 years (M = 15.7%; F = 13.2%). Among hormonal preparations, only children aged 28 days to 2 years (M = 15.3%; F = 12.7%), and those aged 2 to 11 years (M = 13.4%; F = 11.5%) showed a modest difference of use between males and females (Figure 1).

**Figure 2:**
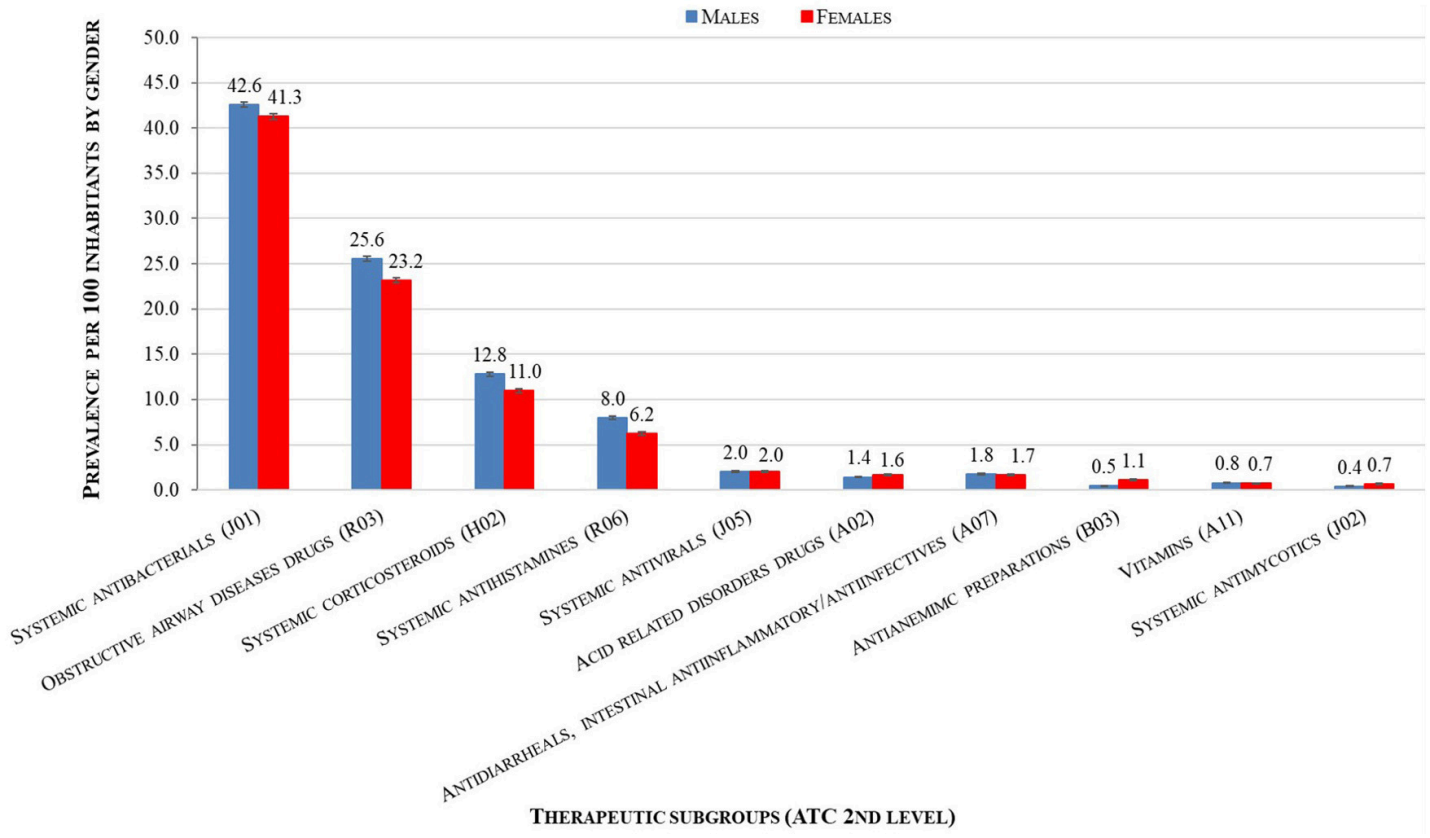
Gender distribution of the prevalence per 100 children of use of most commonly used drug classes in 2015 at therapeutic subgroups (2nd level of ATC).

Looking at therapeutic subgroups (by 2nd level ATC), very small, but statistically significant, differences of prevalence of use was found among boys and girls, irrespective of the age. For example, antibiotics for systemic use (J01^*^) were used in 42.6% of boys and 41.3% of girls, drugs indicated in obstructive airway diseases (R03^*^) in 25.6% of boys and 23.2%, corticosteroids for systemic use (H02^*^) in 12.8% of boys and 11.0% of girls, as well as antihistamines for systemic use (R06^*^) in 8.0% boys and 6.2% of girls (*p* < 0.001) (Figure 2).

Results from the analysis by individual compounds (5th level ATC) belonging to the four commonly prescribed drug classes are described in Table 2. Irrespective of the age-category and gender, amoxicillin/clavulanate in combination was the most used medication (35.3% of pediatric population), followed by beclomethasone (28.4%), cefixime (21.0%), betamethasone (20.2%), azithromycin (15.4%), clarithromycin (14.5%), salbutamol combined with ipratropium (11.1%) or alone (9.5%), cetirizine (8.9%), and amoxicillin (8.5%).

**Table 2:** Distribution^*^ of the 10 most frequently used drugs within each pediatric age group by gender in the year 2015.

		**Any use**			**Recurrent use**		
**Active substance**	**Total children treated (%)**	**Males (%)**	**Females (%)**	***p*-value**	**Males (% of row)**	**Females (% of row)**	***p*-value**
**All ages**	***N* = 100,730**	***N* = 52,513**	***N* = 48,217**	**–**	**–**	**–**	**–**
Amoxicillin/clavulanate	35,551 (35.3)	19,034 (36.2)	16,517 (34.3)	< 0.001	1,878 (9.9)	1,477 (8.9)	0.003
Beclomethasone	28,589 (28.4)	14,931 (28.4)	13,658 (28.3)	0.707	1,276 (8.5)	1,052 (7.7)	0.009
Cefixime	21,187 (21.0)	10,874 (20.7)	10,313 (21.4)	0.008	940 (8.6)	866 (8.4)	0.519
Betamethasone	20,303 (20.2)	11,182 (21.3)	9,121 (18.9)	< 0.001	925 (8.2)	649 (7.1)	0.002
Azithromycin	15,542 (15.4)	8,211 (15.6)	7,331 (15.2)	0.058	656 (7.9)	550 (7.5)	0.257
Clarithromycin	14,330 (14.5)	7,715 (14.7)	6,615 (13.7)	< 0.001	482 (6.2)	380 (5.74)	0.207
Salbutamol/ipratropium	11,169 (11.1)	6,047 (11.5)	5,122 (10.6)	< 0.001	193 (3.1)	136 (2.7)	0.095
Salbutamol	9,579 (9.5)	5,648 (10.8)	3,931 (8.2)	< 0.001	325 (5.7)	216 (5.5)	0.588
Cetirizine	8,943 (8.9)	5,175 (9.9)	3,768 (7.8)	< 0.001	783 (15.1)	498 (13.2)	0.011
Amoxicillin	8,605 (8.5)	4,551 (8.7)	4,054 (8.4)	0.142	265 (5.8)	200 (4.9)	0.069
**Age** ** < 28 days**	***N*** **=** **149**	***N*** **=** **70**	***N*** **=** **79**	**–**	**–**	**–**	**–**
Beclomethasone	58 (38.9)	25 (35.7)	33 (41.8)	0.449	–	–	–
Salbutamol/ipratropium	11 (7.4)	5 (7.1)	6 (7.6)	0.916	–	–	–
Amoxicillin	11 (7.4)	4 (5.7)	7 (8.9)	0.464	–	–	–
Clarithromycin	7 (4.7)	2 (2.9)	5 (6.3)	0.317	–	–	–
Amoxicillin/clavulanate	5 (3.4)	2 (2.9)	3 (3.8)	0.750	–	–	–
Betamethasone	5 (3.4)	4 (5.7)	1 (1.3)	0.132	–	–	–
Budesonide	5 (3.4	3 (4.3)	2 (2.5)	0.553	–	–	–
Cefixime	4 (2.7)	3 (4.3)	1 (1.3)	0.255	–	–	–
Cefaclor	3 (2.0)	2 (2.9)	1 (1.3)	0.490	–	–	–
Fluticasone	1 (0.7)	–	1 (1.3)	–	–	–	–
**Age 28 days –** ** < 2 years**	***N*** **=** **13,521**	***N*** **=** **7,108**	***N*** **=** **6,415**	**–**	**–**	**–**	**–**
Beclomethasone	6,043 (44.7)	3,135 (44.1)	2,908 (45.3)	0.152	275 (8.8)	225 (7.7)	0.145
Amoxicillin/clavulanate	4,518 (33.4)	2,453 (34.5)	2,065 (32.1)	0.004	241 (9.8)	199 (9.6)	0.832
Salbutamol/ipratropium	3,238 (23.9)	1,793 (25.2)	1,445 (22.5)	< 0.001	48 (2.7)	40 (2.8)	0.874
Betamethasone	2,839 (21.0)	1,571 (22.1)	1,268 (19.7)	< 0.001	132 (8.4)	81 (6.4)	0.043
Clarithromycin	2,755 (20.4)	1,528 (21.5)	1,227 (19.1)	< 0.001	93 (6.1)	64 (5.2)	0.327
Cefixime	2,381 (17.6)	1.295 (18.2)	1,086 (16.9)	0.049	103 (8.0)	84 (7.7)	0.843
Amoxicillin	1,726 (12.8)	907 (13.0)	819 (12.7)	0.991	66 (7.3)	44 (5.4)	0.106
Azithromycin	1,688 (12.5)	926 (12.7)	762 (11.8)	0.043	39 (4.2)	34 (4.5)	0.801
Salbutamol	1,482 (11.0)	880 (12.4)	602 (9.3)	< 0.001	37 (4.2)	29 (4.8)	0.574
Budesonide	1,380 (10.2)	782 (11.0)	598 (9.3)	0.001	43 (5.5)	27 (4.5)	0.409
**Age 2–11 years**	***N*** **=** **60,298**	***N*** **=** **31,514**	***N*** **=** **28,784**	–	**–**	**–**	–
Amoxicillin/clavulanate	22,390 (37.1)	11,955 (37.9)	10,435 (36.2)	< 0.001	1,284 (10.7)	1,029 (9.9)	0.031
Beclomethasone	18,252 (30.3)	9,506 (30.2)	8,746 (30.4)	0.556	813 (8.6)	704 (8.0)	0.219
Cefixime	13,719 (22.8)	7,043 (22.3)	6,676 (23.2)	0.013	638 (9.1)	589 (8.8)	0.628
Betamethasone	12,226 (20.3)	6,749 (21.4)	5,477 (19.0)	< 0.001	593 (8.8)	419 (7.7)	0.023
Azithromycin	10,789 (17.9)	5,736 (18.2)	5,053 (17.5)	0.039	472 (8.2)	404 (8.0)	0.658
Clarithromycin	8,562 (14.2)	4,526 (14.4)	4,036 (14.0)	0.232	290 (6.4)	240 (5.9)	0.377
Salbutamol/ipratropium	6,918 (11.5)	3,686 (11.7)	3,232 (11.2)	0.072	119 (3.2)	76 (2.4)	0.028
Salbutamol	6,834 (11.3)	3,974 (12.6)	2,860 (9.9)	< 0.001	223 (5.6)	148 (5.2)	0.432
Cetirizine	6,407 (10.6)	3,696 (11.7)	2,771 (9.4)	< 0.001	561 (15.2)	342 (12.6)	0.004
Budesonide	5,173 (8.6)	2,831 (9.0)	2,342 (8.1)	< 0.001	233 (8.2)	179 (7.6)	0.437
**Age 12–18 years**	***N*** **=** **30,759**	***N*** **=** **16,005**	***N*** **=** **14,754**	–	**–**	**–**	–
Amoxicillin/clavulanate	9,182 (29.9)	4,948 (30.9)	4,234 (28.7)	< 0.001	255 (5.2)	191 (4.5)	0.153
Betamethasone	5,523 (18.0)	3,026 (18.9)	2,497 (16.9)	< 0.001	155 (5.1)	108 (4.3)	0.166
Cefixime	5,429 (17.7)	2,719 (17.0)	2,710 (18.4)	0.002	148 (5.4)	150 (5.5)	0.882
Beclomethasone	4,743 (15.4)	2,521 (15.8)	2,213 (15.0)	0.068	101 (4.0)	58 (2.6)	0.008
Azithromycin	3,252 (10.6)	1,653 (10.3)	1,599 (10.8)	0.146	105 (6.4)	86 (5.4)	0.238
Clarithromycin	3,205 (10.4)	1,784 (11.1)	1,421 (9.6)	< 0.001	67 (3.8)	53 (3.7)	0.969
Amoxicillin	2,389 (7.8)	1,295 (8.1)	1,094 (7.4)	–	43 (3.3)	25 (2.3)	0.13
Cetirizine	2,162 (7.0)	1,252 (7.8)	910 (6.1)	< 0.001	202 (16.1)	135 (14.8)	0.411
Salbutamol	1,386 (4.5)	868 (5.4)	518 (3.5)	< 0.001	49 (5.6)	30 (5.8)	0.909
Salbutamol/ipratropium	1,139 (3.7)	636 (3.9)	503 (3.4)	0.009	11 (1.7)	3 (0.6)	0.085

* *All percentages are calculated on the age-specific number of treated children, either male or female (% of column), except for recurrent use. In this case, the percentages are calculated on the number of children, either male or female, treated with the specific active substance*.

When stratifying by age-category, beclomethasone was found to be the most utilized active substance in the first 2 years of life, in both sexes. The prevalence of beclomethasone use in the youngest age group, that is, children aged less than 28 days (M = 35.7%; F = 41.8%, *p* = 0.449) increased in children aged between 28 days and 2 years (M = 44.1%; F = 45.3%, *p* = 0.152). Thereafter, beyond 2 years of age, amoxicillin/clavulanate in combination was the most commonly used drug, being more commonly used in children aged 2 to 11 years (M = 37.9%; F = 36.2%, *p* < 0.001) compared to children aged 12 to 18 years (M = 30.9%; F = 28.7%, *p* < 0.001).

Regarding gender differences, amoxicillin/clavulanate was more commonly prescribed in boys than girls (M = 36.2%, F = 34.3%, *p* < 0.001), as well as betamethasone (M = 21.3%, F = 18.9%, *p* < 0.001), clarithromycin (M = 14.7%, F = 13.7%, *p* < 0.001), and salbutamol alone or combined with ipratropium (respectively, M = 11.5%, F = 10.6%; and 9.5%; M = 10.8%, F = 8.2%, both *p* < 0.001). On the contrary, cefixime showed modestly but statistically significantly higher prevalence of use in girls than in boys (M = 20.7%, F = 21.4%, *p* = 0.008), while no gender-difference was observed for the overall use of beclomethasone (M = 28.4%, F = 28.3%, *p* = 0.707). When stratifying the analysis by age-category, gender-differences followed a similar trend for all individual compounds analyzed except for cefixime, which was prescribed predominantly among boys until they were 2 years old, with pattern reversing after 2 years of age.

When we analyzed recurrent use (≥3 prescriptions per year) of specific active substances, we found modestly but statistically significantly higher prevalence in boys than girls only for amoxicillin/clavulanate (M = 9.9%, F = 8.9%, *p* = 0.003), betamethasone (M = 8.2%, F = 7.1%, *p* = 0.002), cetirizine (M = 15.1%, F = 13.2%, *p* 0.011) and, contrary to its acute use, for beclomethasone (M = 8.5%, F = 7.7%, *p* = 0.009). The results were confirmed when looking at the highest levels of ATC, such as for antibiotics (M = 51.2%; F = 50.6%, *p* < 0.001), corticosteroids for systemic use (M = 9.2%; F = 7.4%, *p* < 0.001), β_2_-agonist agents (M = 3.6%; F = 2.6%, *p* < 0.001), and their combinations (M = 4.1%; F = 3.4%, *p* < 0.001). Concerning the polytherapy in pediatrics, we found similar proportions of males and females treated concomitantly with at least four different active substance (5,690/52,513 treated boys, 10%; 4,318/48,671 treated girls, 8.9%).

**Figure 3:**
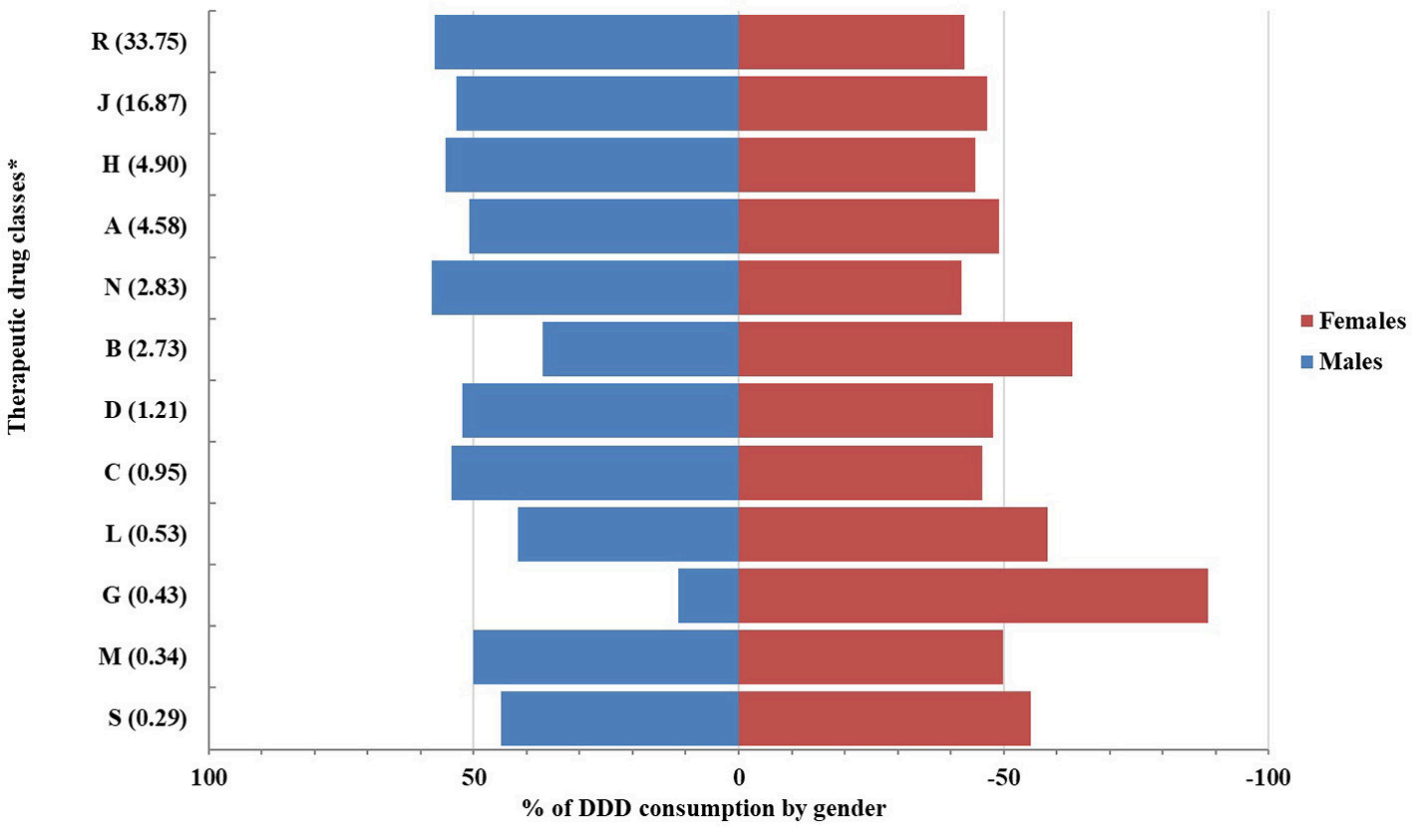
Proportions of drug consumption (by DDD/1,000 persons per day) distributed by gender. ^*^Drug classes by 1st level of ATC (DDD/1,000 persons per day).

In the secondary analysis, the proportion of DDDs/1,000 persons per day was found to be similarly distributed among males and females with some exceptions (Figure 3). A greater proportion of DDDs/1,000 persons per day for medicines belonging to genito-urinary system and sex hormones (ATC: G^*^) or to blood and blood-forming organs agents (ATC: B^*^) was utilized among girls than boys at any age (88 and 66% of DDDs for girls, respectively for G^*^ and B^*^).

## Discussion

This retrospective cohort study is the first to in-depth evaluate gender differences on pediatric drug use. Indeed, to our knowledge there is no other recent work that describes this aspect in Italy, although drug utilization pattern among children has been investigated from previous studies (Cazzato et al., 2001; Vaccheri et al., 2002; Rossignoli et al., 2007; Clavenna and Bonati, 2009b; Clavenna et al., 2009a; Piovani et al., 2013). The main finding from the present study is that there are often small gender differences in drug utilization patterns and, less commonly, large difference between male and female children. The added value of this study performed during a 6-years period of observation (from 2010 to 2015) is that it provides an updated overview of prescribing patterns children/adolescents aged less than 18 years.

Interestingly, a significant decreasing prevalence of overall drug use in children, together with a reduction of the median prescription number, was observed for both sexes over time, most likely due to a decreased prescribing trend of antibiotics. Our results are in line with previous study from Chai et al. where a decreasing trend of medicine presribing among US children seen from 2002 to 2010 was attributed to a reduction of systemic antibiotic market (Chai et al., 2012). It is not clear if the decreased use of antibiotics is correlated to a reduction of infectious diseases but, several previous studies have argued an inappropriate use of antibiotics, leading to serious consequences (Ciofi Degli Atti et al., 2006; De Luca et al., 2016). Indeed, beyond the increasing development of drug-resistant strains of bacteria and viruses which make infections harder to treat (Ventola, 2015), with negative impact even on healthcare costs (Napolitano et al., 2013), profound short- and long-term effects of antibiotics on the diversity and composition of the gut microbiota have recently been associated with the onset of obesity, diabetes, asthma, allergy and autoimmune disease (juvenile rheumatoid arthritis) in later life (Vangay et al., 2015). To promote a conscious use of antibiotics, several initiatives were promoted at national level in the past years, even in Italy, where the Italian Medicines Agency (AIFA) launched campaigns addressing antibiotic use in 2008, 2009, 2010, 2014, and 2015 (AIFA, 2014). Thus, our analyses could provide a positive feedback of changing of antibiotic prescribing habits following such initiatives AIFA.

On increasing the details in drug utilization, i.e., analyzing 1st and 2nd ATC levels rather than any drug use, our results are consistent with previous national and international literature in terms of drug class and age (Cazzato et al., 2001; Rossignoli et al., 2007; Sturkenboom et al., 2008; Clavenna et al., 2009b; Carnovale et al., 2013; Piovani et al., 2013). First of all, the present study confirms that systemic anti-infective agents (mainly, antibiotics for systemic use), respiratory tract drugs (i.e., inhaled corticosteroids, β_2_-agonist agents, and their combinations) and hormonal preparations (i.e., corticosteroids for systemic use) are the most commonly prescribed drugs in the pediatric population studied and that there is a higher prevalence of drug use among children aged 1 month to 2 years, as found in Northern Italy. The similarity with results from Northern Italy study is even more evident when exploring the top ten individual drugs used in children. Interestingly, the prevalence of amoxicillin/clavulanate use and amoxicillin use at any age is lower in our study as compared to the study from Piovani et al. (35.3 vs. 41.6% for amoxicillin/clavulanate; 8.7 vs. 18.2%, for amoxicillin, respectively) despite an increase of the prevalence of other antibiotics (i.e., cefixime: 21.0 vs. 13.8%, respectively) (Piovani et al., 2013). Although this prescription trend could suggest fluctuations of prevalence of some bacteria more than others across regions, thus justifying different use of single antibiotic at regional level, we cannot exclude a potential variability of prescribing/dispensing habits. Likewise, we found that the prescription of beclomethasone was greater compared to other Italian studies [28.4 vs. 23.5% by (Piovani et al., 2013)]. Considering that, to our knowledge, no evidence exists with regard to a higher prevalence of asthma among children living in the area of LHU of Caserta, it is possible that there is over-use of inhaled corticosteroids, in particular for beclomethasone and flunisolide, especially for the treatment of upper respiratory tract infections. However, reasons for this geographic heterogeneity need to be further investigated.

Concerning gender differences in drug utilization in more detail, anti-infective agents, respiratory drugs and hormonal preparations were often significantly although modestly different between genders; stratifying these results by age group highlighted the differences. Drugs belonging to these classes, as well as to antibiotics and anti-asthmatic drugs specifically, were prescribed slightly but consistently more frequently among boys than girls. The slightly higher frequency of use among boys compared to girls was in line with previously published findings, although in the present study, the trend persisted among all age groups while Clavenna et al. found that the trend reversed in adolescents, with girls being prescribed more drugs than boys (Sturkenboom et al., 2008; Clavenna et al., 2009b). The reason behind this is not clear, since there is no apparent gender-related pharmacological requirement or medical risk which is higher in males compared to females. Potential explanations for gender-related drug utilization patterns could be found looking at the indication of use. On the other hands, the incidence and prevalence of some childhood diseases may differ across gender, or male children may be at a higher risk of asthma than females or certain infections than girls (Subbarao et al., 2009; Tekgul et al., 2012).

Gender-differences are also confirmed by subgroup analysis on the recurrent use, defined as at least 3 prescriptions in a year, for amoxicillin/clavulanate, more commonly prescribed in boys aged 2–11 years than in girls. Of note, concerning the recurrent use of any antibiotic, we found out that half of treated pediatric population, whether boys or girls, received at least three prescriptions of any antibiotic in 1 year. This common recurrent use of antibiotics may be due to antibiotic resistance, but over-use of antibiotics can also cause antibiotic resistance.

Interestingly, gender differences were found for the recurrent, but not for acute, use of inhaled beclomethasone, consistently with previous publication (Sturkenboom et al., 2008). In line with the pediatric indication of beclomethasone (SmPC of beclomethasone diproprionate), the higher prevalence of beclomethasone use in boys could suggest a greater recurrence of asthma episodes/exacerbation, thus confirming the relationship between drug intake and prevalence of such disease in boys. On the other hand, previous pharmacoepidemiological studies suggested that use of anti-asthmatic drugs, particularly inhaled corticosteroids, is not a good indicator of asthma (Sen et al., 2011; Bianchi et al., 2016). Indeed, it has been documented the off-label use of anti-asthmatic medicines, together with antibiotics, very often prescribed for symptomatic treatment of upper respiratory tract infections, such as cough, pharyngotonsillitis, rhinitis, otitis media, and sinusitis (Bianchi et al., 2011). Likewise, the number of non-asthmatic children and adolescents treated with inhaled corticosteroids is ten times higher in Italy than in the UK or in the Netherlands (Sen et al., 2011). Moreover, as reported in our study, irrespective of gender, beclomethasone is the most utilized medicine in the 2 first years, focusing the con concerns on its correct use. As beclomethasone has been listed by EMA among medicine of interest for research on use and safety in pediatrics (European Medicines Agency, 2016), our data can be useful by providing a pharmacoepidemiological background/context for future working assessing the impact of drug use that can support regulatory body decisions.

In the present study, DDD consumption seems to be equally distributed among males and females with some exceptions. Since DDD consumption usually indicates a chronic treatment, a consumption of gynecological-antiseptic drugs and sex hormones (G01^*^, G02^*^, G03^*^), with gender-related indications, was expected to be more pronounced in girls (OsMed, 2015), as well as greater consumption of iron preparations (B03^*^), usually prescribed for conditions due to blood loss associated with female reproductive system, such dysmenorrhea (De Andrade Cairo et al., 2014). Overlapping results were obtained from multi-database study across Italy, Netherlands and UK (Sturkenboom et al., 2008). We suggest that the consistency found between our data and Italian data would suggest the generalizability of our results at national level.

Results from this study specifically performed in pediatric population differ from drug utilization patterns among adults, which suggest a higher prevalence of medicine use among women, probably because of their higher median age leading to a greater probability of being to be exposed to multiple therapies (Zopf et al., 2009). This further suggests that drug exposure information among adults cannot be generalized to children, highlighting the importance of our results.

Finally, safety data in children from ADR spontaneous reporting system found a higher frequency of ADR in boys than in girls till 12 year of age. Indeed, our findings suggesting a slightly higher use of medications among males compared to females may be one potential explaination for the gender difference in ADRs among pre-adolocents. Thereafter, the drug utilization trend is not consistent with the ADR pattern, which increases for adolescent girls. This discrepancy could be due to the use among this population of medicines which cannot be traced using the data source used in this study (e.g., ibuprofen or other non-steroidal anti-inflammatory drugs, dispended as over-the-counter medication).

The main strength of this study is its novelty in examining gender differences in prescribing in a pediatric setting in great detail. Another important strength is the large population size, as a result of which the majority of the children in a wide catchment area of Southern Italy was included in the study. In addition, since the data source used for the study consists of a claims database containing information derived from routine clinical practice, the results of the study are likewise considered to represent actual prescribing practices. However, this study also has some limitations. Drug exposure among neonate outpatients is likely to have been underestimated, as children may be registered with a pediatrician later 1 month after birth, resulting in the missing data for this age group. This study suggests the need to capture neonatal drug exposure from other data sources, such as birth registries and hospital databases with mother-children linkage. Another limitation is the possibility of underestimating the use of medicine even in older children through these data sources. Indeed, the cost of antibiotic and anti-asthmatic drugs is at least partially reimbursed by National Health Service. However, given the relatively cheap cost of some antibiotics and the parents' attitude to consult to private pediatricians, the confounding role of out-of-pocket purchase of antibiotics, which is not traceable using the study data source, cannot be excluded purchase of antibiotics, ultimately resulting in a possible underestimation of overall prevalence of antibiotic use in children. Further investigations are needed to address this issue.

## Conclusion

In a large scale of pediatric outpatients from Southern Italy, no major differences in drug use by gender were observed. In general, however, it seems that the most commonly used therapeutic drug groups in pediatrics (i.e., antibiotics, medicines for respiratory tract disease and corticosteroids for systemic use) are prescribed modestly but consistently to larger extent in males than females. This is in contrast with drug use in adults, were women seem be prescribed more medicines than men. Irrespective of gender, the higher prevalence of inhaled corticosteroids together with antibiotics requires careful consideration about their potential inappropriate use for symptomatic treatment of upper respiratory tract infections. Interestingly, this study shows also a decreasing trend in antibiotic use in pediatrics in recent years in contrast to previous real world Italian investigations based on outdated data, probably as a result of campaign antibiotic resistance taken at national and regional level.

## Author Contributions

CF and GT conceptualized, designed and coordinated the study. CF and JS carried out the analyses and wrote the manuscript. VI, CS, and GS designed the data collection instruments, collected data and finalized the analyses. MT provided data collection. AC and FR supervised the study. CF, JS, VI, CS, GS, GT, FR, and AC critically reviewed the manuscript for intellectual content and approved the final manuscript as submitted.

### Conflict of Interest Statement

The authors declare that the research was conducted in the absence of any commercial or financial relationships that could be construed as a potential conflict of interest.
